# Study on the antidepressant activity of (2R,6R; 2S,6S)-Hydroxynorketamine (HNK) and its derivatives

**DOI:** 10.1016/j.clinsp.2024.100435

**Published:** 2024-07-13

**Authors:** Dongdong Zang, Xuemei Yang, Hao Wang, Zhenxing Li, Yanjun Ma, Jianxi Liu, Xi Mei, Shupeng Li, Jinxing Feng, Xin Shi, Zhen Tan

**Affiliations:** a Department of Neonatology, Shenzhen Children's Hospital, Shenzhen City, Guangdong Province, China; b Health Management Center, Shenzhen University General Hospital, Shenzhen University Clinical Medical Academy, Shenzhen University, Shenzhen City, Guangdong Province, China; c Department of Neurosurgery, Shenzhen People's Hospital (The Second Clinical Medical College, Jinan University; The First Affiliated Hospital, Southern University of Science and Technology), Shenzhen City, Guangdong Province, China; d Guangdong Engineering Technological Research Center for Nervous anatomy and Related Clinical Applications, Shenzhen City, Guangdong Province, China; e Shenzhen Ruijian Biotechnology Co., LTD, Shenzhen City, Guangdong Province, China; f Zhuhai Pengkun Biomedicine Technology Co. LTD, Zhuhai City, Guangdong Province, China; g State Key Laboratory of Oncogenomics, School of Chemical Biology and Biotechnology, Peking University, Shenzhen City, Guangdong Province, China; h Department of Neurosurgery, Shenzhen University General Hospital and Shenzhen University Clinical Medical Academy Centre, Shenzhen University, Shenzhen City, Guangdong Province, China

**Keywords:** Hydroxynorketamine and its Derivatives, Forced Swimming Test, Behavioral Sensitization Test, Conditioned Place Preference Test, Antidepressant

## Abstract

**Objective:**

This study mainly explores (2R,6R; 2S,6S)-HNK and its compounds whether there are antidepressant effects.

**Methods:**

Four HNK compounds were obtained from 2-(Chlorophenyl) Cyclopentylmethanone. Forced swimming test, locomotor sensitization test, and conditioned location preference test were used to screen the antidepressant activity of the synthesized target compounds.

**Results:**

In the case of 10 mg HNK treatment, compared with saline, the immobile time of mice in the HNK group, I5 group and I6 group at 1 h and 7 days had statistical significance. In the case of 10 mg HNK treatment, compared with saline, the immobile time of compound C and D groups in the glass cylinder area was significantly different. In the locomotor sensitization test, the movement distance of compound C and D groups on day 15 and day 7 mice increased significantly compared with the first day. In the conditioned place preference experiment, compound C and compound D induced conditioned place preference in mice compared with the Veh group.

**Conclusion:**

The results of the forced swimming test, locomotor sensitization test, and conditioned location preference test showed that compounds C and D may have certain anti-depressant activity. However, HNK exerts a rapid and significant antidepressant effect within 1 week, but the duration is short.

## Highlights

• In the case of 10 mg HNK treatment, compared with saline, the immobile time of compound C and D groups in the glass cylinder area was significantly different.
• In the locomotor sensitization test, the movement distance of compound C and D groups on day 15 and day 7 mice increased significantly compared with the first day.
• In the conditioned place preference experiment, compound C and compound D induced conditioned place preference in mice compared with the Veh group.

## Background

Depression disorder is a common mental illness. Surveys in recent years show that the lifetime prevalence rate of depression disorder is about 6.8 % and the annual prevalence rate is about 3.6 %, which affects the health and life of more than 89 million people [1]. Currently, commonly used antidepressants on the market mainly act on the monoaminergic system, including Monoamine Oxidase Inhibitors (MAOIs), Tricyclic Antidepressants (TCAs), Tetracyclicanti Depressants (TeCAs), selective 5-Hydroxytryptamine (5-HT), Selective 5-HT Reuptake Inhibition (SSRIs), and so on. The biggest drawback of these drugs is that they are slow to work. At present, antidepressants work by altering the metabolic processes and biological efficacy of monoamine neurotransmitters. It has been widely accepted that monoaminergic neurotransmitter-based antidepressants are effective, but there are still some problems, such as many adverse reactions, limited efficacy, and apparent late onset of action [2]. Therefore, the search for effective, rapid and safe antidepressant targets and treatment methods has aroused extensive interest from clinical and basic researchers.

Ketamine was first synthesized in 1962, used in humans in 1965, and officially approved for clinical use by the FDA in 1970 [3]. Ketamine was included in the list of Essential medicines of the World Health Organization until 1985 [4]. The effect of ketamine on improving acute pain has been widely confirmed, and studies have also confirmed that ketamine can also improve chronic pain, especially neuropathic pain [5,6]. In the last 10 years, with the study of ketamine usage and dosage, and its anti-inflammatory, antidepressant, neuroprotective, and analgesic effects have been discovered, the medical community's interest in ketamine is surging again. The use of ketamine as an antidepressant has a sophisticated molecular mechanism. Glutamate, as an excitatory neurotransmitter in the central nervous system, plays a role in regulating synaptic plasticity. Functional brain imaging revealed simultaneous inhibition of function, size, and synaptic plasticity in the prefrontal and hippocampus during depressive episodes. Ketamine is a derivative of PCP. After entering the human body, ketamine binds to a variety of receptors in the central nervous system, including glutamate receptor N-Methyl-D-Aspartate Receptor (NMDAR), AMPA-type ionotropic glutamate Receptor (AMPAR), and opioid receptor, etc. Signaling pathways such as Tropomysin-related Kinase B (TrkB) and its ligand Brain-Derived Neurotrophic Factor (BDNF) are involved [7]. In 2000, Berman et al. first reported that more than 50 % of patients with a single intravenous subanesthetic dose of ketamine (0.5 mg/kg) had a more than 50 % reduction in the Hamilton Depression Scale score within 72 h. In recent years, a number of animal and clinical studies have further confirmed the antidepressant effects of ketamine. Ketamine has also been used as anesthesia for electroconvulsive therapy in depressed patients [8]

According to the patent “Application of (2R,6R)-hydroxynormethamine, (S)-dehydromethylketamine, and other stereoisomeric dehydrogenation and hydroxylation metabolites of (R,S)-ketamine in the treatment of depression and neuropathic pain”, central nervous system side effects were found to be related to the activity of (R,S)-ketamine against NMDA receptors. Based on ketamine, (2R,6R; 2S,6S)-Hydroxynorketamine (HNK), a compound that is not active against NMDA receptors, is synthesized, thus avoiding possible side effects. The present experimental study found that (2R,6R; 2S,6S)-HNK do not last long after administration, and are basically inactive within 1 week, which severely limits the long-term effects hoped for in the treatment of depression. At the same time, this study also found that the application of HNK causes adverse effects on patients. So structural modification of (2R,6R; 2S,6S)-HNK to obtain longer potency and less addictive drugs has great therapeutic potential. It can be seen that ketamine and its compounds are promising to be the lead compounds of drugs derived from natural products and have the prospect of further study. This study mainly explores (2R,6R; 2S,6S)-HNK and its compounds.

## Materials and methods

### Experimental animals

C57BL/6J male mice of 8‒12 weeks, weighing 18‒22 g, were reared at 22 ± 1 °C, humidity of (50 % ± 10 %), and light time from 8:00 to 20:00. The mice were fed and drank freely, and adapted to the experimental environment for at least 2‒3 days. All experiments were conducted from 8:00 to 16:00.

### Pharmacological experimental methods

#### Preparation and administration method of drug

All drugs were dissolved with 0.5 % sodium carboxymethylcellulose (2R,6R; 2S,6S)-HNK and its compounds and administered by gavage.

#### Forced swimming test (FST)

Grouping methods: 1) Mice were divided into 4 groups with 10 mice in each group. Mice were given 1 mg (A), 10 mg (B) and 30 mg (C) HNK by intragastric administration, and their immobility time was measured 1 h and 7 days later.

2) Mice were divided into 4 groups with 10 mice in each group. Mice were given 10 mg and 30 mg HNK, compound C and compound D by intragastric administration, and their immobility time was measured.

Methods: Mice were transferred to the laboratory 1 h before FST. The test was conducted in normal light conditions and monitored by a digital camera. During the test, mice were placed in a transparent glass cylinder (28.5 cm high and 14 cm in diameter) filled with 20 cm of water (23 ± 1 °C). On the first day, the mice were trained for 6 min and then removed from the cylinder. On the second day, mice were treated with saline, HNK, I5, I6, C, and D at different time intervals. During the last 4 min of the entire 6-min swim test, the immobility time was recorded via the Noldus system's EthoVision XT (Noldus, Netherlands), defined as passive floating with no movement other than those necessary to keep the head on the water. The water was replaced after every two or three tests. After FST, the mice were removed from the water and dried under a red light.

#### Behavioral sensitization test

Grouping method: The mice were randomly divided into 4 groups with 10 mice in each group. The groups were vehicle (veh), I6, C, and D (5.0, 10.0, 30.0 mg/kg).

Experimental methods: Animal spontaneous movement infrared analysis system, composed of spontaneous movement box, infrared probe device, and data acquisition system. The self-actuating box is 40 × 40 × 65 cm, sound-proof, light-proof, and ventilated. The activity of mice was recorded by infrared probe and the spontaneous activity was calculated. To test the effect of the drug on the spontaneous activity of mice, the mice were given intragastric administration once a day in the morning for 7 days. Subsequently, the drug was discontinued for 7 days (no treatment). On day 15, mice were given the drug intragastric. The spontaneous activities of days 1, 7 and 15 were measured within 1 h immediately after administration.

#### Conditional position preference (CPP) test

The experiment adopts a bias procedure, which is divided into three stages: pre-test, training, and test. During the experiment, environmental conditions such as light, tone and smell in the box are ensured to be consistent.

Pre-test: Days 1‒3, the shuttle door of the box was opened, all mice were injected with subcutaneous saline and put into the intermediate box and allowed to move freely in the box for 15 min, once a day, for consecutive 3 days. The residence time of mice in the black and white boxes was recorded to determine the natural preference tendency. Mice were trained with an unnatural preference box.

Training: On days 4‒9, with the shuttle door closed, mice were randomly divided into Veh, mor, I6, C, D (5.0, 10.0, 30.0 mg/kg) and mor (10 mg/kg) with 10 mice in each group. In the morning of singular days, all mice were first given normal saline by intragastric administration and immediately placed in the black box for 45 min. In the afternoon of singular days, Veh, mor, I6, C, and D (5.0, 10.0, 30.0 mg/kg) were given intragastatically and immediately placed in the white box for 45 min. Even days were practiced in reverse order. The training interval in the afternoon was more than 6 h, and the training time was fixed every day. The training lasted for 6d, with 10 mice in each group, 150 in total.

Test: On day 10: the shuttle door was opened, the mice were placed into the intermediate box and ran freely. Meanwhile, the time of mice staying in the white box within 15 min was recorded.

### Statistical analysis

Statistical analyses were performed using SPSS 20.0 software (SPSS Inc., Chicago, IL). Shapiro-Wilk tests were used to determine the normality of data. Measurements were displayed as mean ± standard deviation (X ± S), and group comparisons were made using *t*-tests (two groups) or ANOVA tests (three or more groups) for data on continuous variables in a normal distribution; data on continuous variables in a skewed distribution were analyzed using the Mann-Whitney *U* test (two groups) or the Kruskal-Wallis (three or more groups). Multiple group comparisons were examined using Tukey's multiple comparison test for data. Count data were expressed as frequencies and ratios and Chi-Square or Fisher's exact test was used; p < 0.05 was considered statistically significant. Data were plotted using GraphPad Prism 8 (GraphPad, San Diego, CA).

## Results

### FST

**Fig. 1:**
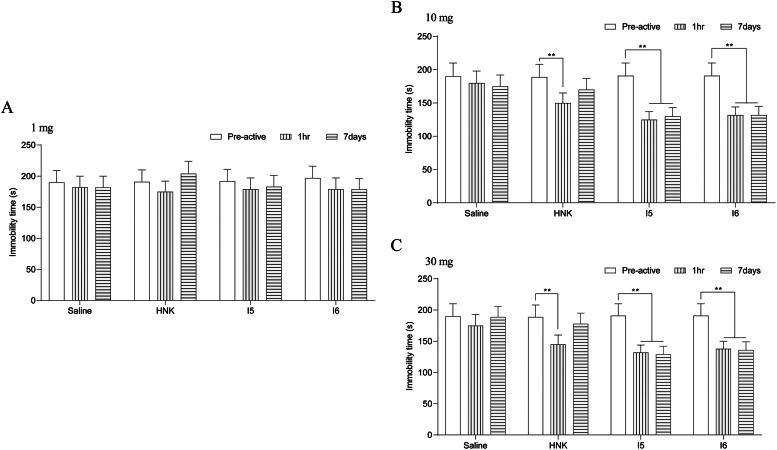
Antidepressant effects of different doses within 7 days.

To compare the FST results in the HNK, I5 and I6 groups, the Kruskal-Wallis test was used for FST comparison. Pharmacological experiments showed no significant difference observed in FST in the 4 groups under normal light conditions (p > 0.05). Mice in groups 1 mg HNK, I5, and I6 were given intragastric administration, and the results of FST showed that compared with the saline group, the immobility time of the three groups after 1 h and 7 d had no significant difference observed (p > 0.05, Fig. 1A). After treatment with 10 mg HNK group, compared with saline, the immobility time of mice was significantly decreased in 10 mg HNK group, I5 group, and I6 group at 1 h (p < 0.01), and increased at day 7 (p > 0.05). The immobility time of mice was statistically significant between-group I5 and group I6 after 1 h and 7 days (F = 4.333, 2, 2, p < 0.05, Fig. 1B). Compared with saline, the immobility time of mice in 30 mg HNK group, I5 group, and I6 group was significantly decreased at 1 h (p < 0.01), and increased at 7 days (p > 0.05). The immobility time in the I5 group and I6 group was statistically significant after 1 h and 7 days (F = 1.857, 2, 2, p < 0.01, Fig. 1C).

### Antidepressant effects of compounds C and D

**Fig. 2:**
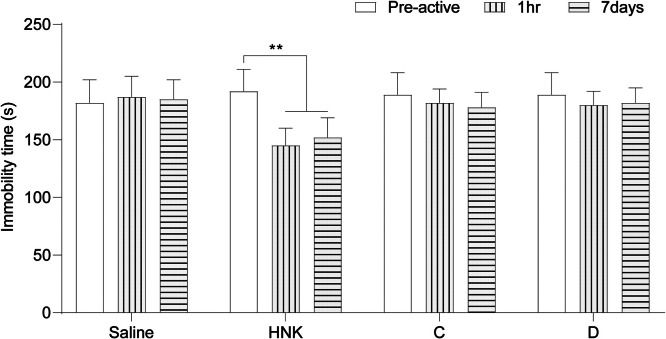
Antidepressant effects of compounds C and D.

Pharmacological experiments showed that there was no significant difference observed in FST under normal light conditions (p > 0.05). FST results showed that the immobility time of mice in 10 mg and 30 mg HNK groups was significantly decreased compared with the saline group, with statistical significance (p < 0.05). However, compared with 10 mg HNK, the immobility time was increased under the condition of 30 mg HNK treatment. Compared with saline, the immobility time of mice treated with 10 mg or 30 mg of compounds C and D showed no significant difference observed (both p > 0.05) (Fig. 2).

### Effects of compounds on behavioral sensitization in mice

**Fig. 3:**
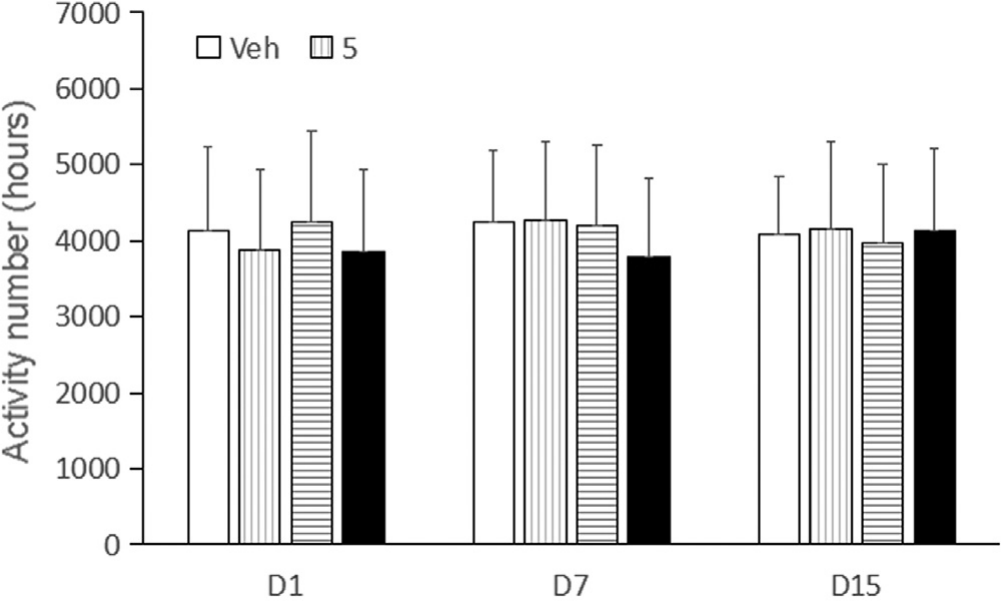
Compound I5 showed no sensitization.

**Fig. 4:**
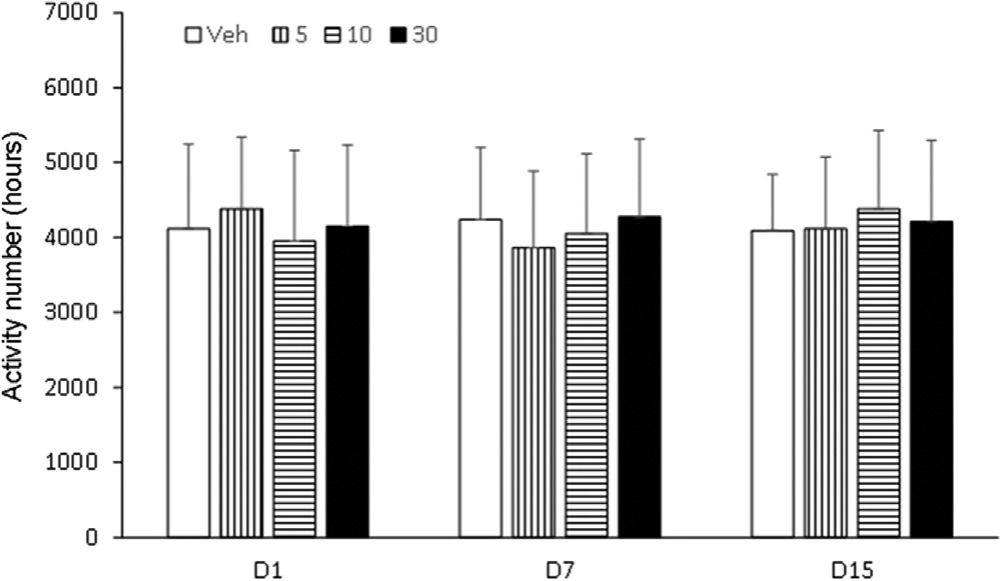
Compound I6 showed no sensitization.

**Fig. 5:**
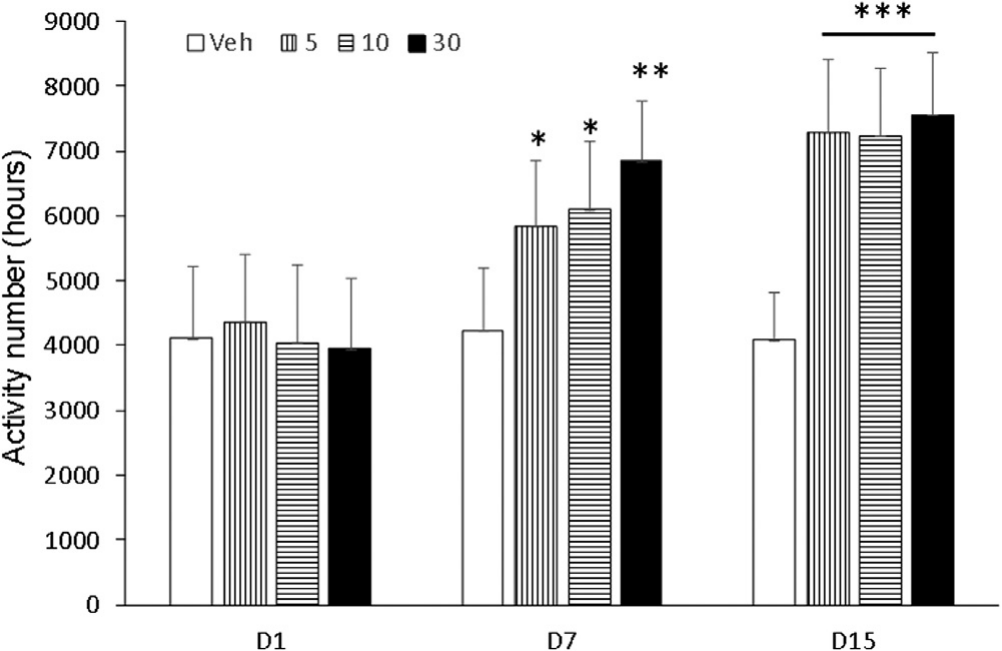
Compound C showed behavioral sensitization.

**Fig. 6:**
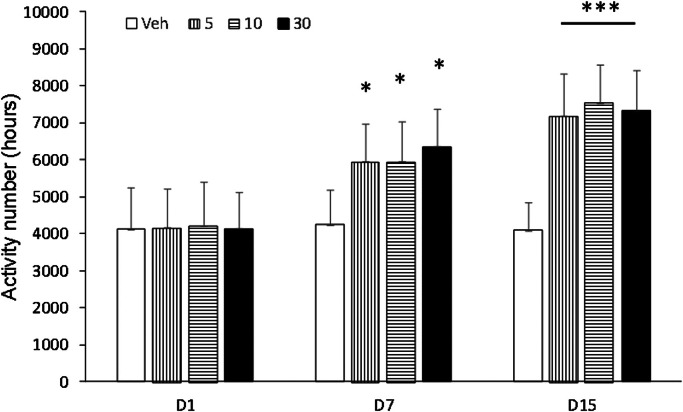
Compound D showed behavioral sensitization.

Behavioral sensitization in mice showed that the motion number of mice in Veh group on day 7 and day 15 had no significant difference compared with day 1 (p > 0.05). Compared with Veh group, motion times of I5 and I6 groups (5.0 mg/kg, 10.0 mg/kg and 30.0 mg/kg) on days 1, 7 and 15 were not statistically significant (p > 0.05, Fig. 3, Fig. 4, Fig. 5). Compared with the Veh group, the motion times of compound D and Compound C at 5.0 mg/kg, 10.0 mg/kg and 30.0 mg/kg on days 1, 7 and 15 were not statistically significant (p > 0.05, Fig. 6).

### CPP test

**Fig. 7:**
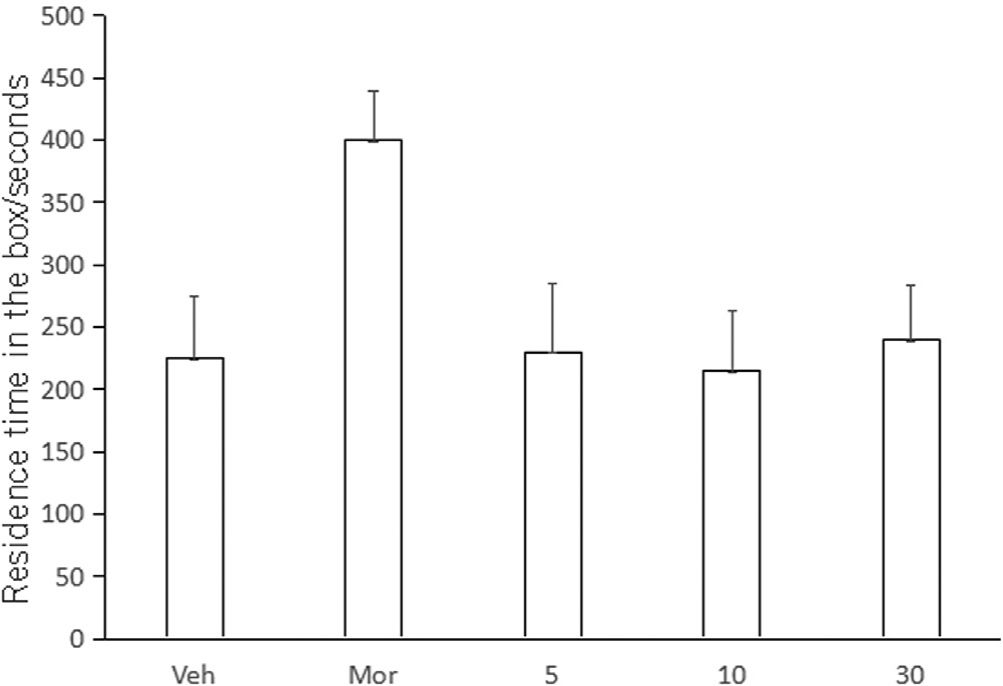
I5 showed no CPP in mice.

**Fig. 8:**
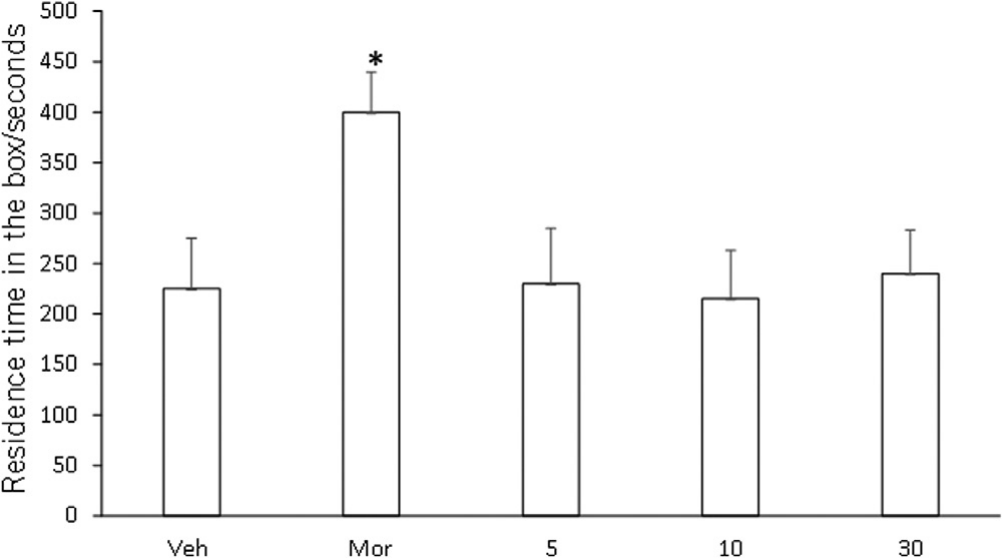
I6 showed no CPP in mice.

**Fig. 9:**
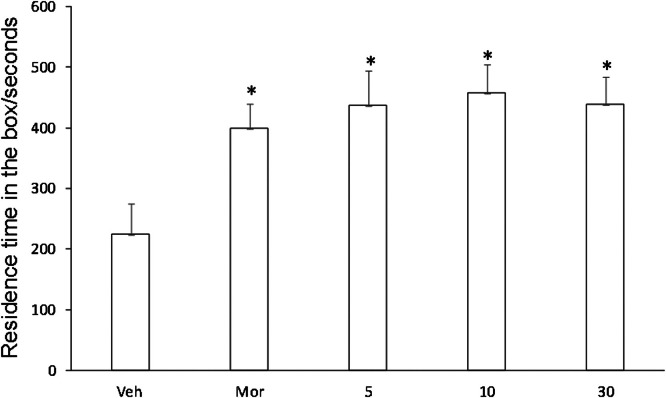
Compound C induces CPP.

**Fig. 10:**
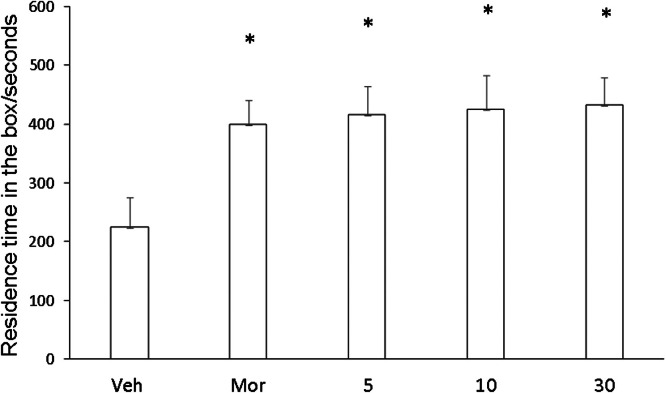
Compound D induced CPP.

In order to compare the CPP results of Veh, Compound C, and Compound D groups, Kruskal-Wallis test was used for CPP comparison. Compared with Veh group, CPP was formed in mice given compound C (5.0, 10.0, 30.0 mg/kg), and CPP formation was significantly induced by compound D, and CPP score was significantly different from that in Veh group (p < 0.01, Fig. 7). Compared with Veh group, CPP was also formed in mice given compound C (5.0, 10.0, 30.0 mg/kg), with no significant difference in CPP score (p < 0.01, Fig. 8). No CPP was formed in I5 and I6, and CPP scores were not significantly different from those in Veh group (p > 0.05), but CPP could be induced in mor group (Fig. 9, Fig. 10).

## Discussion

Since 2000, the rapid antidepressant effects of ketamine and its enantiomers (S)-Ketamine and (R)-Ketamine have received attention [9]. Ketamine can not only be used as a free anesthetic for general anesthesia, but also as a painkiller for acute, chronic, cancer, perioperative pain, and the pain of critically ill patients [10]. As well as being a free anesthetic for general anesthesia, ketamine is also used to treat acute, chronic, cancerous, or perioperative pain, as well as pain associated with critical illness[10]. and has become an abused recreational drug [11]. Ketamine exhibits antidepressant and antisuicide properties,[8]. demonstrating its potential as a treatment for depression and creating hope for new antidepressants. According to “Application of (2R,6R)-hydroxynormethamine, (S)-dehydromethylketamine, and other stereoisomeric dehydrogenation and hydroxylation metabolites of (R,S)-ketamine in the treatment of depression and neuropathic pain”, (2R,6R; 2S,6S)-HNK, a compound that is not active against NMDA receptors, is synthesized based on ketamine, thus avoiding possible side effects. (2R,6R; 2S,6S)-HNK showed little long-term effect after administration, with almost no activity within a week, severely limiting the long-term effect on depression.

The experimental model of FST in mice provides a compression environment. The immobile state exhibited by mice in the experiment reflects a state known as “behavioral despair and distortion” or “failure to adapt to a pressurized environment” [12]. This classical model is sensitive to most antidepressants and is therefore widely used to screen for such drugs. This model was used to measure the immobility time in mice treated with 1 mg, 10 mg and 30 mg HNK after 1 h and 7 days. FST results all showed that the immobility time of mice in the HNK treatment group was decreased and that in the 10 mg and 30 mg HNK, I5, and I6 groups was significantly different from that in the saline control group. It is worth noting that, the 10 mg and 30 mg HNK groups showed significant antidepressant effects after 1 h, but the duration was short, with almost no activity within 1 week. I5 and I6 were active for over a week, suggesting that I5 and I6 have sustained and stable antidepressant effects. Moreover, there are significant behavioral differences after long-term treatment of I5 and I6 at different doses (1 mg, 10 mg, 30 mg). In addition, I5 and I6 both showed stable and strong antidepressant effects compared with HNK in the experiment. It is consistent with Berman's findings that rapid administration of HNK results in an antidepressant effect within 2 hours [13,14]. Whether the substance basis of its antidepressant effect is related to the high content is worthy of further study. At the same time, I5 and I6 showed antidepressant effects compared with compounds C and D in the experiment. I5, I6, and compounds C and D all had certain antidepressant activity. Compounds I5 and I6 were the most active, as they are rich in -OH, followed by HNK and compounds C and D. Structurally, the more hydroxyl groups contained in the B ring, the better the activity seems to be, which is consistent with literature reports [15]. Halogenated compounds also show some antidepressant activity in the order 2-Cl > 2-Br > 2-F > I. The same halogen has different activities depending on where it is substituted. The conclusion obtained in this study is consistent with that of Zhao DH et al [16]. Tripathi et al. introduced 3-hydroxy-3-phenacyloxindole analogues, which showed better inhibitory activity on monoamine oxidase A than on monoamine oxidase B [17]. In 2021, Kumar et al. introduced indole-3-piperazinyl derivatives. Derivatives with R2 as biphenylbenzyl showed high inhibitory activity, with IC50 values less than 1 μmoL/L, among which compound 21 had the strongest inhibitory activity, with IC50 value of 0.11 μmoL/L, 193 times that of type B. In addition, when the interval length of methylene group n is 3, the phenyl substituted compound 22 also shows strong inhibitory activity and selectivity, its IC50 value is 0. 14 μmoL/L, and it has no activity against type B. Compound 21 is expected to be developed as a therapeutic candidate for depression [18]. The results showed that the structure change of the compound resulted in different degrees of improvement in its activity, which proved that the transformation of hydroxyl esterification was helpful to improve its pharmacological activity. The study of (HNK) and its derivatives provides a theoretical basis for finding new, safe and less side effects antidepressants.

Experimental and behavioral sensitization models of high activity due to addictive substances are two commonly used animal models [19]. The brain becomes hypersensitive to drugs and drug-related stimuli after repeated and intermittent exposure to addictive substances, resulting in pathological drug cravings [20]. Drugs that are administered repeatedly and intermittently lead to behavioral sensitization, which refers to the effects of those drugs (such as morphine, amphetamine, cocaine, nicotine, alcohol, etc.) on behavior. The behavioral sensitization model has been widely used as an animal model to evaluate the potential of drug psycho-dependence. Behavioral sensitization is one of the characteristics of drug addiction and plays an important role in compulsive drug use and relapse behaviors [21]. A large number of research results have reached the same conclusion [22,23]. Behavioral sensitivities are divided into two phases, the formation and manifestation phases. Drugs that can affect behavioral sensitization may have therapeutic effects on drug addiction. Rodent locomotor vigilance is manifested by behavioral sensitization, which refers to psychomotor enhancement following repeated, intermittent exposure to drugs. It is generally accepted that behavioral sensitization to addictive substances depends on adaptive changes in the functioning of the DA system in the mesolimbic limbic cortex. The ventral tegmental area and prefrontal cortex are mainly responsible for the formation of behavioral sensitization, and the manifestation of sensitization is mainly NAc. The results of this study showed that behavioral sensitization was induced by intermittent gavage of compound C and compound D in mice, and the behavioral sensitization was related to the dosages of compound C and compound D. But the specific pharmacological mechanism is unclear.

It is also possible that ketamine triggers intracellular signaling and synaptic changes that produce a rapid antidepressant effect, which then triggers downstream signaling for sustained effects. Similarly, the early signals required to prevent the acute antidepressant effects of ketamine block the sustained effects, and these findings suggest that signaling and protein synthesis lead to subsequent changes in neuronal structure, function, and connectivity, and ultimately to long-term behavioral effects [24]. However, there are still relevant questions that need further investigation: the underlying molecular and cellular mechanisms behind the results of carrying out the experiment, and how these mechanisms relate to antidepressant activity.

The model of COO is to combine an environmental stimulus with a drug as conditioned reinforcement, allowing the animal to establish a relationship between the environmental stimulus and the drug, thus observing the animal's preference and measuring the reinforcing effect of the drug combination. It manifests itself as a reward effect, and the underlying principle is the theory of classical conditioning. CPP subsides at certain intervals, or the animal's preference effect can be restored through regression training, the use of environmental stimuli, and small doses of drugs. Therefore, CPP is a reliable animal model for evaluating the effects of addictive drugs and a direct means to study addiction-related conditioning. It has been reported that sodium depletion training can effectively dissipate CPP induced by psychoactive drugs [25]. This study also adopted this CPP paradigm and found that most mice developed CPP after treatment with compounds C and D. This is consistent with the results reported that HNK can successfully induce the CPP paradigm [26]. However, unlike HNK, compounds C and D were not sufficient to cause a preference or aversion to the drug-matching environment in mice [27]. In conclusion, HNK induced by gavage plays an important role in maladaptive behavior. However, until now, the specific effects of compounds C and D need further study.

## Conclusion

Compounds C and D had significant effects on the formation of behavioral sensitization, FST and CPP tests in mice. This suggests that compounds C and D have certain antidepressant activity. This study provides a preliminary basis for the screening of ketamine derivative antidepressants in the future and has important significance for the prescription of rapid onset and good activity of antidepressants.

## Availability of data and material

Data is available from the corresponding author on request.

## Ethical statement

All animal experiments complied with the ARRIVE guidelines and performed in accordance with the National Institutes of Health Guide for the Care and Use of Laboratory Animals. The experiments were approved by the Institutional Animal Care and Use Committee of Shenzhen Children's Hospital (n° GCC2023016).

## Authors' contributions

Dongdong Zang, Xuemei Yang and Hao Wang designed the research study. Zhenxing Li, Yanjun Ma and Jianxi Liu performed the research. Shupeng Li and Xi Mei provided help and advice. Zhenxing Li, Jinxing Feng, Xin Shi and Zhen Tan analyzed the data. Dongdong Zang, Xuemei Yang and Hao Wang wrote the manuscript. Jinxing Feng, Xin Shi and Zhen Tan reviewed and edited the manuscript. All authors contributed to editorial changes in the manuscript. All authors read and approved the final manuscript.

## Funding

1. Supported by Guangdong High-level Hospital Construction Fund; Basic research of Shenzhen Science and Technology Plan Project (General Program: JCYJ20220530155611026)
2. Supported by Sanming Project of Medicine in Shenzhen “Multidisciplinary epilepsy diagnosis and treatment team of Prof. Wang Yuping from Xuanwu Hospital Capital Medical University” (SZSM202003006).
3. Shenzhen Health Elite Talent Project (n 2021XKQ193).
4. Shenzhen Fund for Guangdong Provincial High-level Clinical Key specialties (No.SZXK035).
5. The Sanming Project of Medicine in Shenzhen (Grant no. SZSM202311027).
6. Supported by Guangdong High-level Hospital Construction Fund.
7. Clinical key specialty construction project of Guangdong Province.

## Declaration of competing interest

The authors declare no conflicts of interest.

## Appendix Supplementary materials

Image, application 1
