# Supplementary material for: Study on the antidepressant activity of (2R,6R; 2S,6S)-Hydroxynorketamine (HNK) and its derivatives
Source: Clinics (Sao Paulo). 2024 Jul 13;79:100435. doi: 10.1016/j.clinsp.2024.100435 (PMC11301192; doi:10.1016/j.clinsp.2024.100435)
Supplement: Supplementary file 1 [file mmc1.docx]

**CLINICS-D-24-00241_ Supplementary Material**

**Material Supplementary**

| **2.2.1 Synthesis of (2R,6R)-6-HNK** |
| --- |
| ① In double-mouth or three-mouth bottles, 50g magnesium powder was added and initiated, a solution (119.2g cyclopentyl bromide and THF mixture) was added and refluxed for 2‒4h to prepare 1.6 moL/L cyclopentyl grignard reagent. The mixture of THF and o-chlorobenzonitrile (50.0g) was added to 840 mg CuBr, and the cyclopentyl grignard reagent (1.6 moL/L, 280 mL) was added by drops under the condition of ice bath, refluxed for 1h, and cooled to room temperature. Then, 100 mL water was added, and 200 mL 15% dilute sulphuric acid solution was supplemented and stirred overnight. THF was spin-dried, treated with EA extraction, dried, and passed through silica gel column to obtain 60g compound A, 2-chlorophenyl cyclopentyl methanone, with 80% yield. |
| ② 20g of compound A (96 mmoL) was dissolved in 400 mL EA, added with copper bromide (54g, 242 mmoL), heated and refluxed for 3h, and cooled to room temperature. The solid insoluble substrate was filtered by diatomite, and the filter residue was washed with dichloromethane and concentrated with the filtrate to obtain the yellow oil-like compound B, 2-chlorophenyl (1-broMocyclopentyl). Compound B pure product was obtained by silica gel column with a yield of 80%. |
| ③ Ammonia gas was added into 200 mL ammonia water until saturated, and compound B (10g) was added and stirred for 24h. Compound C was precipitated, filtered, and dried to obtain brown solid compound C (7g) with a yield of 70%. |
| ④ Compound C (5g) was dissolved in dry THF and exposed to HCl gas until the solution pH was 1, and the solution was spin dried to obtain solid carbonate. The solid carbonate was added into a single-outlet flask, placed in an oil bath at 190°C under nitrogen protection for about 20 min, cooled to room temperature, and neutralized with sodium bicarbonate saturated solution. After extraction with DCM, the compound D norketamine HNK racemite (2.9g) was obtained by concentration, with a yield of 75%. H NMR (400 MHz, CDCl3): δ7.67 (dd, J=7.8, 1.5 Hz, 1H), 7.37‒7.32 (m, 2H), 7.25 (m, 1H), 2.78‒2.71 (m, 1H), 2.61 (m, 1H), 2.51‒2.43 (m, 1H), 2.08‒2.0 (m, 1H), 1.88‒1.63 (m, 4H) |
| ⑤ Compound D (1.11g, 5 mmoL) was dissolved in 2 mL methanol, then L-tartric acid (2.5 mmoL) was added, stirred for 1h, dropped into 10 mL acetone, and then crystallized and filtrated to obtain L-tartaric acid crystals. The crystals after continued recrystallization for 3 times were neutralized in sodium bicarbonate solution, and extracted with EA to obtain optically pure compound E, (R)-norketamine (165 mg). The optical purity was 98.3% and the yield was 15% after analysis by chiral HPLC. Chiral HPLC detection procedure: Compound E (1 mg) and the control racemite compound D (1 mg) were dissolved in 1 mL ethanol, and then placed on Agilent 1260-A high performance liquid chromatograph for normal phase evenness analysis. The chromatographic column was Chiralcel-AD-H (4.6×250 mm), with mobile phase A: (n-hexane + 0.1% diethylamine), mobile phase B (ethanol + 0.1% diethylamine), A:B = 40:60, and flow rate at 1 mL/min. Compound E: The retention time of R configuration was 6.8 min, and the corresponding retention time of the isomer S configuration was 5.3 min. |
| ⑥ Compound E (2.23g, 10 mmoL) was added into 60 mL THF, and triethylamine (2.7 mL, 20 mmoL) and Boc_2_O (3.3g, 15 mmoL) were added and refluxed for 6h. Compound F was obtained by silica gel column after cooling and spinning dry. The yield of compound F was 90%. 1H NMR (400 MHz, CDCl3): δ 7.81 (d, J = 8.1 Hz, 1H), 7.40-7.28 (m, 2H), 7.24‒7.12 (m, 1H), 6.57 (s, 1H), 3.82 (d, J = 14.4 Hz, 1H), 2.45‒2.36 (m, 1H), 2.28 (m, 1H), 2.04 (m, 1H), 1.89‒1.56 (m, 4H), 1.27 (s, 9H). 13C NMR (100 MHz, CDCl3):δ 207.9, 152.3, 134.5, 132.6, 130.3, 130.0, 128.3, 125.2, 78.0, 66.1, 38.5, 37.4, 29.7, 27.2, 20.9. |
| ⑦ Compound F (2.91g, 9 mmoL) was added into 60 mL dry THF and then cooled to -78°C under the protection of argon gas. Then, 5 mL HMPA was added, and then 2 M LDA THF solution (12 mL, 24 mmoL) was slowly added and stirred for 30‒40 min. Then it was slowly heated to -30°C and stirred for 1h, and then cooled to -78°C, and added to trimethylchlorosilane (2.6g, 24 mmoL). Then it was slowly heated to -50°C and stirred for 3h, followed by treatment with saturated ammonium chloride solution to room temperature. THF was concentrated and extracted with EA. The organic phase was dried with anhydrous Na_2_SO_4_, spin-dried, and vacuum-dried. The obtained oil was dissolved in 100 mL anhydrous DCM and cooled to -15°C. Under the protection of argon, mCPBA (2.5g, 11 mmoL) was added and stirred for 1h to room temperature, 50 mL DCM was added and stirred for another 1h, and then saturated sodium thiosulfate and sodium bicarbonate solution (1:1) were added. After extraction with DCM, the solution was spin dried and vacuum dried, and the obtained oil was dissolved in 100 mL THF and then cooled to -5°C. Tetrabutylammonium fluoride compounds C and D (3g, 11.4 mmoL) were added and stirred for 30 min. Then, with saturated NaHCO_3_ solution, the solution was extracted with EA, spin-dried, vacuum-dried, and passed through the silica gel column to collect the compound G 1.92g (yield 65%). 1H NMR (400 MHz, CDCl3): δ 7.81 (d, J = 7.8 Hz, 1H), 7.34 (m, 2H), 7.24 (m, 1H), 6.60 (s, 1H), 4.12 (dd, J = 11.7, 6.8 Hz, 1H), 3.87 (d, J = 14.4 Hz, 1H), 3.38 (m,1H), 2.36 (m, 1H), 1.74 (m, 2H), 1.68‒1.57 (m, 1H), 1.55‒1.40 (m, 1H), 1.30 (s, 9H). 13C NMR (100 MHz, CDCl3): 209.8, 153.2, 134.1, 133.6, 131.3, 130.8, 129.5, 126.2, 79.3, 72.2, 66.5, 40.3, 38.7, 28.1, 19.4. |
| ⑧ Compound G (680 mg) was dissolved in 5 mL dry THF, exposed to gas HCl at room temperature to saturation, and stirred for 4h. Then, 20 mL dried diethyl ether was added, and the precipitated crystal was filtrated to collect 520 mg compound H (2R,6R)-6-HNK, with the yield of 95%. 1H NMR (400 MHz, CD3OD): δ 7.85 (m, 1H), 7.65‒7.51 (m, 3H), 4.28 (m, 1H), 3.19 (m, 1H), 2.30 (m, 1H), 1.81‒1.72 (m, 2H), 1.64-1.51 (m, 2H) |
| ⑨ Compound G (170 mg, 0.5 mmoL) was dissolved in 3 mL dried THF, followed by the addition of dried triethylamine (0.28 mL, 2 mmoL) and then 3-furanyl chloride (130 mg, 1 mmoL) in an ice bath. The compound was warmed to room temperature within 1h and stirred overnight, and then sodium bicarbonate solution was added. It was then extracted with EA, dried by spinning, vacuum-dried, and passed through silica gel column to obtain 173 mg compound H6 N-Boc-(2R,6R)-6-(3-furan) methanoyl norketamine, with a yield of 80%. 1H NMR (400 MHz, CDCl3): 8.11 (br, 1H), 7.81‒7.79 (brs, 1H), 7.42‒7.28 (m, 4H), 6.67 (brs,1H), 6.63 (m, 1H), 5.35-5.31 (m, 1H), 3.92‒3.87 (m, 1H), 2.39-2.33 (m, 1H), 1.94‒1.74 (m, 4H), 1.30 (brs, 9H). 13C NMR (100 MHz, CDCl3): 202.5, 168.5, 161.4, 153.3, 148.4, 143.8, 134.3, 133.7, 131.4, 131.1, 129.8, 126.3, 118.5, 109.1,79.4, 73.5, 67.2, 38.5, 35.8, 28.2, 19.8. |
| Compound H6 (170 mg) was dissolved in 3 mL dry THF, exposed to HCl to saturation at room temperature, and stirred for 4h. Then 15‒20 mL dried diethyl ether was added to the precipitate crystals, and 124 mg compound I6 (2R,6R)-6-(3-furan) methanoyl norketamine hydrochloride was obtained by vacuum filtration, with a yield of 86%. 1H NMR (400 MHz, CD3OD): 9.02 (brs, 3H), 8.09 (s,1H), 7.79 (s, 1H), 7.43-7.40 (m, 4H), 6.75 (br, 1H), 5.39 (m, 1H), 3.76‒3.54 (m, 1H), 2.41‒2.32 (m, 2H), 2.10‒1.93 (m, 3H)13C NMR (100 MHz, CD3OD): δ200.2, 168.5, 161.3, 148.8, 144.0, 136.9, 134.8, 132.1, 131.5, 129.3, 128.4, 118.5, 110.0, 73.6, 68.2, 37.3, 3 5.1, 19.5. |
|  |
| **2.3 Compound synthesis** |
| **2.3.1 Synthesis of HNK** |
| 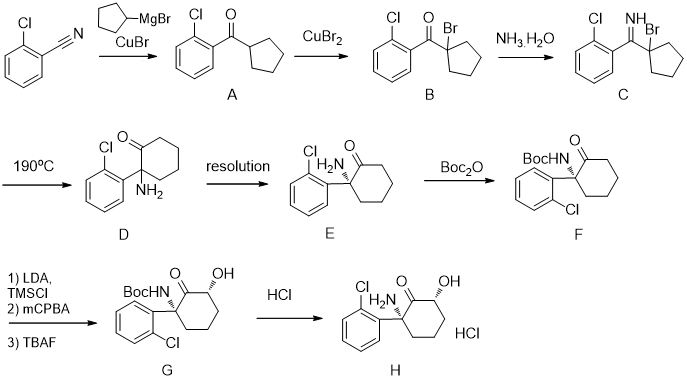 |
|  |
| **2.3.2 Synthesis of compound I6** |
|  |
|  |
| **2.3.3 Synthesis of compound C\D** |
|  |
|  |
